# “When I Go There, I Feel Like I Can Be Myself.” Exploring Programme Theory within the Wave Project Surf Therapy Intervention

**DOI:** 10.3390/ijerph16122159

**Published:** 2019-06-18

**Authors:** Jamie Marshall, Paul Kelly, Ailsa Niven

**Affiliations:** 1 School of Applied Sciences, Edinburgh Napier University, 9 Sighthill Court, Edinburgh EH11 4BN, UK; 2 Physical Activity for Health Research Centre, Institute for Sport, Physical Education and Health Sciences The University of Edinburgh Holyrood Road, Edinburgh EH8 8AQ, UK; p.kelly@ed.ac.uk (P.K.); ailsa.niven@ed.ac.uk (A.N.)

**Keywords:** qualitative research, grounded theory, mental health, programme theory, surf therapy, physical activity

## Abstract

Mental health issues in young people are a priority for health and social care. Surf therapy is an innovative intervention that may help address this health burden globally. While increasing evidence demonstrates the effectiveness of surf therapy, there has been limited exploration as to how it achieves its outcomes. Such theoretical exploration is important for service optimisation, monitoring and proliferation. This research aimed to adopt, for the first time, a rigorous grounded theory approach to explore underlying programme theory within the Wave Project surf therapy intervention. Participants (*n* = 22, 14 males and 8 females; mean age = 14 years, SD = 3.5, range 8–23) were interviewed about their intervention experiences. Data were analysed through constant comparative analysis and memo writing. Two core categories reflected mediators by which surf therapy may achieve its outcomes: “Self-Selected Pacing and Progression While Surfing” and “Creation of Emotional and Physical Safe Space at Beach”. Three antecedent (linking known inputs to core categories) and three consequent categories (linking core categories to associated outputs) were also identified. These demonstrate theorised pathways from known inputs to associated outcomes within the intervention. These important findings provide plausible evidence on how to optimise the Wave Project’s delivery in tackling mental health burden.

## 1. Introduction

Mental health is defined as a state of well-being in which every individual realises his or her own potential, can cope with the normal stresses of life, can work productively and fruitfully, and is able to contribute to his or her community [1]. Mental health problems are one of the main causes for overall disease burden worldwide accounting for 21.2% of years lived with disability [2]. In England, for example, 1 in 6 people report experiencing a common mental health problem (such as anxiety and depression) in any given week [3]. There is a need for accessible evidence-based interventions to combat these challenges. 

Physical activity has a strong evidence base for promoting and treating mental health [4,5]. Specifically, physical activity has been demonstrated to have a positive impact on depression [6], anxiety [7], post-traumatic stress disorder (PTSD) [8] and well-being [9]. Physical activity interventions that use surfing as a vehicle to achieve positive change (including enhanced mental health), have been termed as ‘surf therapy.’ The recently formed International Surf Therapy Organisation has highlighted how iterations of surf therapy exist within health, social and developmental settings. Studies focused on surf therapy, have primarily prioritised testing and demonstrating effectiveness [10,11,12,13,14,15]. This focus has been necessitated by the sector’s emergent nature alongside the need for intervention funding and sustainability by primarily charitable organisations. There is a growing body of evidence to demonstrate that surf therapy is effective in improving comparable mental health outcomes across different populations in a wide range of contexts including vulnerable young people [12,13,14], disability [14], and military veterans [10,15]. Findings from these studies have demonstrated a strong association between surf therapy and positive mental health outcomes. However, this evidence base still has methodological limitations, and requires further investigation. 

**Figure 1:**
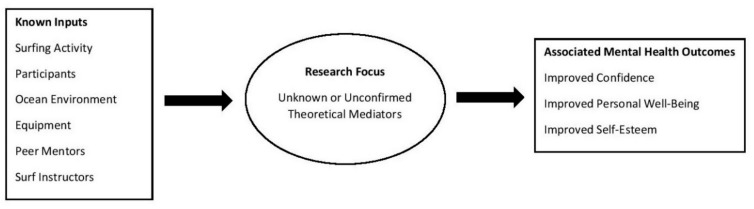
Preliminary logic model.

Although there is evidence that surf therapy can be effective in enhancing mental health, limited research has considered how surf therapy and other blue space therapies may achieve their effects. Indeed, a recent systematic review examining the potential of “blue spaces” for health and well-being highlighted that understanding mechanisms of change is a priority [16]. Developing a programme theory as to how surf therapy works would be beneficial to guide research and evaluation. Programme theory is a comprehensive understanding of how a programme or intervention is supposed to work [17]. A programme theory maps the pathways from intervention inputs to outcomes, with pathways being broken down into theoretical mediators [18]. One of the best ways to present programme theory and mediators remains visualisation through the use of a logic model [19]. Figure 1 illustrates a preliminary logic model for how surf therapy leads to positive mental health outcomes. As illustrated in Figure 1, the known inputs for this iteration of surf therapy are the ocean environment, surf instructors, peer mentors and surf equipment. Meanwhile, the associated positive mental health outcomes for the intervention composed of improved personal well-being, improved confidence and improved self-esteem. 

A limited number of studies have considered potential theoretical mediators relevant to surf therapy. Emerging research into natural ‘blue space,’ other than surf therapy, suggests a potential link between the environment in which surfing occurs with improvements to wellbeing [16,20,21,22,23] and therefore may be a relevant theoretical mediator. In explaining positive findings from effectiveness research, some researchers have speculated that surf therapy may be effective due to its social elements, and that it offers opportunities for respite and to build self-efficacy [10,12]. More targeted empirical explanations are evident from two studies utilising dialogical narrative analysis to explore the experiences of ex-military participants experiencing PTSD who participated in the same UK-based surf therapy intervention [24,25]. Findings from participant observation and life history interviews, suggested that peer relationships and the sense of respite fostered by surfing were important contributors to the programme’s effectiveness. These qualitative studies make an important contribution to the field of surf therapy. However, the participants in these studies were military veterans and it cannot be assumed that these findings can directly translate to other groups, such as young people. A grounded theory approach allows for theoretical mediators to be identified specifically within this context through either the confirmation of theory coherent with other ‘blue space’ and physical activity paradigms or the generation of new theory. Therefore, the rationale for this study is that potential theoretical mediators for surf therapy have not been empirically studied in this population group and context.

As shown in Figure 1, there is a current lack of understanding as to the pathways from known inputs to associated outcomes. Although findings from other physical activity and ‘blue space’ interventions may provide some insight into the potential mediators of mental health outcomes, these findings are not specific to surf therapy and further research is warranted. 

Understanding the theoretical mediators of change and mapping the programme theory is important for enhancing delivery, quality control and monitoring [17]. An articulated programme theory also offers insight and direction for effective proliferation, transfer and scaling of projects in broader contexts and settings [17]. Consideration of theoretical mediator is also important within the development of an evidence base because identifying plausible and testable mediators adds strength to claims of effectiveness [26].

The focus of this study is on understanding how participating in an established surf therapy programme may be beneficial for young people experiencing mental health difficulties. Drawing from the participants’ experiences, the study aims to develop a programme theory that demonstrates the key theoretical mediators that lead from accepted programme inputs to mental health outcomes. Such a model may be used to inform theoretical discussion, service improvement and wider intervention comparison.

## 2. Materials and Methods

### 2.1. Theoretical Framework

A constructivist, grounded theory study design [27] provided the ontological framework for data collection. A grounded approach is most appropriate where there is a lack of theoretical understanding to explain social processes and human behaviour [28], as is the case with surf therapy. This framework aligns with the aim of this study in exploring the theoretical mediators within surf therapy programme theory, all the time remaining based or “grounded” in participant experience. While there exist multiple iterations of grounded theory, the identifying aspects in this project included concurrent data collection and analysis, constant comparison, memo writing, a theoretical sample and continuation of data collection until data saturation was reached [27,29]. Such a method was carried out in a pragmatic manner to allow for theory generation derived from data collected in the practical, ‘real world’ setting [30] of surf therapy.

### 2.2. The Wave Project

The context for this research was the Wave Project, a UK-based charity that utilised surf therapy to support young people facing mental health challenges or social isolation. The Wave Project was founded in 2012 and is primarily funded through partnership with the National Health Service and charitable trust funding grants alongside private donations. The charity has developed a programme of surf therapy that combines the act of surfing with peer mentoring and support utilising local volunteers to achieve its outcomes. Peer mentoring is focused on enabling participation challenges participants may face and celebrating their achievements. Two to three hours of training are provided for new volunteers on how to best to support participants. The programme is currently delivered to c.1200 young people annually across eleven sites in the UK: Scotland, Scarborough, South Wales, North Devon, South Devon, North Cornwall, South Cornwall, Mid Cornwall, Dorset, Brighton and the Isle of Wight. Professionals refer young people to the service who are facing, or are at risk of, mental health challenges, social deprivation and social isolation. These professionals work for organisations such as the Child and Adolescent Mental Health Service which is provided by the National Health Service, the local authority departments such as social and foster care, schools and other mental health charities and organisations. Young people undertake a six-week course, attending weekly for two- to three-hour sessions of surf therapy. Upon completion, participants are encouraged to join a regular opt-in surf club that provides a continuation of surf therapy post the focused six-week intervention. Programme evaluation is consistently positive and this is supported with some non-controlled empirical data [12]. 

### 2.3. Ethics

Ethical approval was granted by the University of Edinburgh Moray House Ethics Committee on 01/07/2017 (ethics ID 343). Appropriate background checks were undertaken on the researcher and disclosure procedures put in place. Written consent was obtained for all participants alongside parents/guardians where appropriate.

### 2.4. Sample

**Figure 2:**
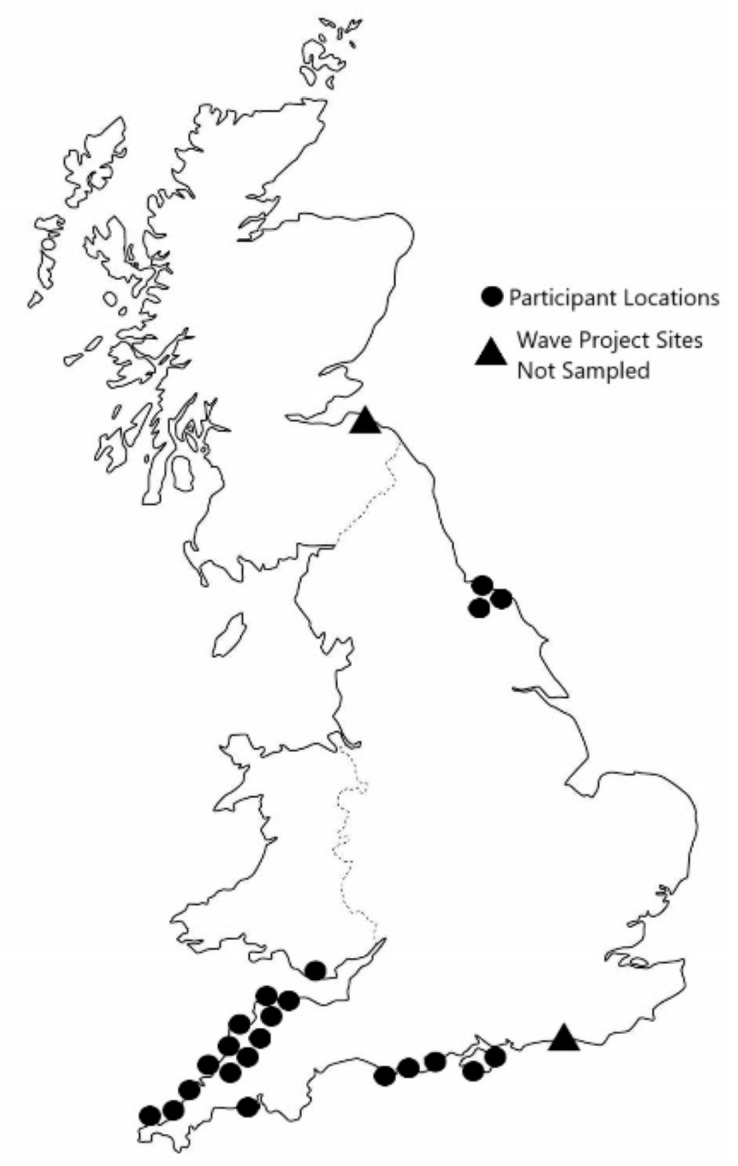
Location of interview sites including sites not sampled.

A purposive theoretical sample [27] was utilised to enable a grounded approach to the research questions. Young people (under 24) who had participated in the Wave Project intervention and surf club for longer than 6 months and were currently active members were invited to partake in the research through Wave Project gatekeepers. Participants were recruited from 9 of the 11 Wave Project sites across the United Kingdom. One site was not sampled due to logistical challenges associated with a change of project management at time of sampling. The project site in Scotland was not sampled due to the lead author working in the program located there, and the subsequent potential power imbalances that could be present within data collection. Twenty-two young people (14 males and 8 females; mean age = 14 years, SD = 3.5, range 8–23) participated in interviews from across England and Wales. Figure 2 demonstrates the geographical spread of young people interviewed. Participant mental health challenges and reasons for referral were not disclosed to the researcher as they were not deemed necessary for the investigation.

### 2.5. Data Collection 

Interviews were conducted between June 2017 and February 2018, with a mean interview time of 16 min and a range of 7 to 32 min (SD = 7.71 min). Seven interviews were conducted via telephone or internet teleconferencing for logistical reasons, and all remaining interviews were conducted in person at locations determined by participants. These locations primarily consisted of participants’ homes or at the beaches where the interventions were sited. Participants were given the opportunity to be interviewed with an additional person present to support them. Nearly a third (31%) took this option, and participants typically chose parents and siblings to be present. These processes enabled a more equal power-sharing situation between interviewer and participant. 

### 2.6. Interview Schedule

Interviews were semi-structured and opened with a very broad question about participant experiences at the Wave Project. The remainder of the interview consisted of core open-ended and non-leading questions that would allow for exploration of participants’ experiences. Further prompts that allowed for elicitation of relevant experiences, while resonance was achieved through clarification prompts to make sure all respondent meanings were truly understood [27]. The initial schedule was developed to ensure thorough exploration of all the processes related to the intervention. This schedule was piloted through the initial few interviews before being further refined (see Supplementary Materials). New questions continued to be added in later data collection to explore emergent categories aligning with grounded theories’ iterative and non-linear approach [27]. 

All 22 interviews were recorded with an Evistr L53 portable MP3 recorder and then transcribed verbatim. Transcription was carried out by a professional transcription service, and the primary researcher subsequently compared the transcripts with the recordings to ensure accuracy and as part of the initial coding process. Analysis was conducted by the lead-researcher in an iterative, emergent and non-linear manner based on constant comparison between participant data [31]. Coding was carried out in three stages with initial, intermediate and advanced coding being utilised throughout the analytical process in line with established grounded theory practice [27,32]. Different coding methods were used for differing reasons throughout analysis (Table 1). A three-stage coding method allowed for extrapolation of individual processes into a wider programme theory and eventual identification of core, antecedent and consequent categories. The final stage of the analytical process also involved mapping of participant pathways from known inputs to associated outcomes to understand directionality and relationships between categories. The coding of transcripts was combined with constant memo writing and reflection throughout the process to map the analytical process. 

Upon completion of 18 interviews, new codes were not being generated and a robust programme theory was identified indicating possible theoretical saturation. Theoretical saturation is the saturation of the properties of a theoretical category involving not just sampling, but the analytical stage as well [33]. Four further interviews were conducted, with new experiences and perspectives described indicating diversity and depth of data. The new data did not add to the emergent programme theory which further indicated saturation. 

### 2.7. Reflexivity

Integral to a grounded approach was reflective practice, i.e., the recognition of the researcher as a participant within research. Reflective practice was not only integral to credibility, rigour and transparency within this research, but is an ethical imperative [34]. The Alvesson and Skölberg [35] model was utilised to offer a framework for reflective practice. Reflective memo writing occurred throughout the process to enable awareness of personal preconceptions, bolster credibility and supplement analysis [27]. An example of reflective practice was the decision to not sample from the Scottish Wave Project site. Due to the researcher’s pre-existing relationship as an intervention leader, research at this site was precluded due to ethical complications related to power relationships [34]. The intersubjective dynamics of being perceived to be at the head of the very intervention under discussion would not have allowed participants to offer public accounts of their experiences. This illustrates one example reflective practice with similar processes carried out at every stage of the project.

## 3. Results

### Core Categories and Programme Theory

**Figure 3:**
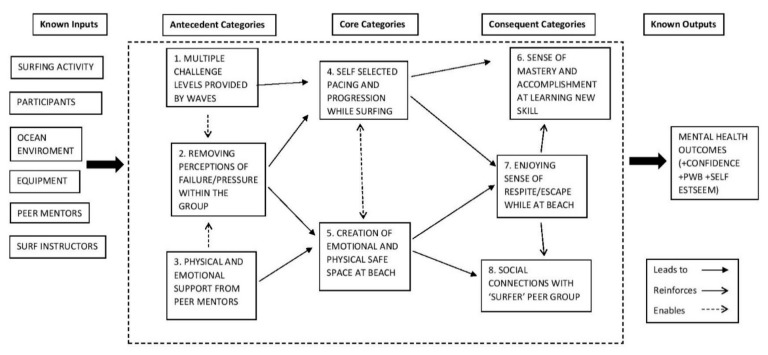
Logic model **s** howing mechanisms of action.

Figure 3 is a logic model that illustrates the programme theory that was constructed from the research findings. The figure illustrates theoretical pathways from known inputs (i.e., ocean environment/volunteers) through Antecedent Categories, Core Categories and Consequent Categories to known outputs. At the centre of these pathways were the two Core Categories; Self Selected Pacing and Progression While Surfing and Creation of Emotional and Physical Safe Space at the Beach which seemed fundamental to intervention functioning. Antecedent Categories highlighted how these Core Categories were achieved. Consequent Categories unpacked, primarily through existing theory, how Core Categories facilitated associated mental health outcomes. Each category will be considered in turn to highlight their content and links with the participants’ experiences. All participant experiences are reported under pseudonyms to protect anonymity. 

Antecedent Categories

1. Multiple Challenge Levels Provided by the Waves

Participants frequently identified the different opportunities and levels for participation enabled by the nature of the surf environment itself.

*“So, in the first lesson, I think we stuck quite shallow, really shallow, and very small waves, which helped quite a lot. Then, I progressed slowly onto the bigger ones.”*

Dan, 16

Within the one beach environment there existed different kind of waves to suit different levels of competency, confidence and physical capability. On any given day of surf, entry level waves are available as shallow as shin depth, while wading a bit further out to waist depth would enable surfers to experience a bit more power and a longer wave. Further out still is ‘out the back’ or beyond the breakers where waves will be available for the most advanced or confident surfers.

*“Starting off with the smaller ones, I kept on working my way up. Medium ones are quite difficult. Eventually, I get used to them then I selected a bigger one. They really worked, so I went on a few more medium ones, started working my way up. I kept on going bigger and bigger.”*

James, 14

**Figure 4:**
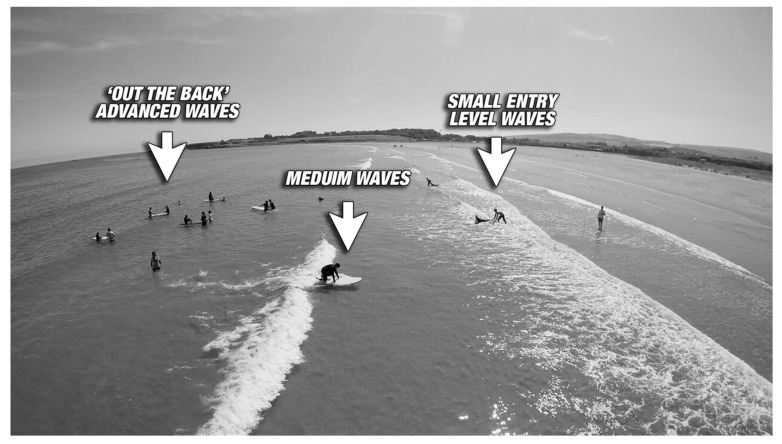
Visualisation of different waves at beach.

The availability of different wave opportunities enabled groups with mixed abilities, and furthermore enabled participants to progress at their own pace. Figure 4 highlights these different opportunities. 

2. Removing Perceptions of Failure/Pressure Within the Group

Throughout data collection, it was frequently reported how the intervention fostered a culture without perceptions of failure or pressure.

*“When I was out there, if I got some of it wrong, or anything, I didn’t get told off. It was just to have fun. They weren’t bothered about if you could do it or not. They just wanted you to relax and have fun.”*

Dan, 16

The removal of concepts of failure and pressure to perform supported participants who may otherwise not feel able to partake in a such a social and potentially performance-orientated environment.

*“There’s no forcing people to do stuff they’re not comfortable with.”*

Ashley, 13

The emphasis on minimising pressure and perceptions of failure presents a unique approach to the delivery of what is at its core a skill-based intervention.

3. Physical and Emotional Support from Peer Mentors

Participants frequently cited the importance of peer support received from volunteer surf mentors to their experiences at the intervention both socially and in terms of achievement.

*“The volunteers were really chatty; every time someone caught a wave they’d cheer and clap.”*

Nicole 18

This support often took the form of encouragement and positive reinforcement relating to individuals’ participation and achievements. Another individual highlighted the celebration of their achievements.

*“Everyone cheers and claps stuff when you catch a wave. It’s so lovely.”*

Jessica, 23

Support also took the form of supporting participants when they may have felt uncomfortable or nervous. One participant highlighted how the adult support was integral to their participation.

*“I’m most nervous about the waves, but when I’m in the waves with an adult helping me, I might feel more comfortable.”*

David, 8

Core Categories

4. Self-Selected Pacing and Progression While Surfing

Autonomy specifically related to pacing and progression was frequently brought up as an integral part of participant experience. One participant highlighted how their own “slow” pace was facilitated at the intervention.

*“Like just go at my own pace which is quite slowly and that was no problem which was so helpful.”*

Jessica, 23

Being able to progress at one’s own pace, whether fast or slow, appeared critical to subsequent achievement, enjoyment and adherence throughout intervention delivery. Participants directly linked this to improvements in confidence achieved through this autonomy of pacing and the role it played in supporting consequent categories related to mastery/achievement.

*“Well, I started on the sand or not going in the water. Then I started out shuffling about in the water, doing things. Then I think I just tried on the surfboard, and I think I started to like it, then confidence grew over me.”*

Stephanie, 12

5. Creation of Emotional and Physical Safe Space at Beach

Participants identified the beach and the intervention as a safe space where they felt comfortable participating in the surfing within a welcoming social environment.

*“It’s a really nice safe space for a young person to go and join in and be allowed to have fun and feel safe and welcome.”*

Nicole, 18

These feelings of comfort and safety were often contrasted with descriptions of negative emotions and feelings within wider life, for example one individual compared feelings at the intervention with their anxiety and self-consciousness in day-to-day life.

*“I have really bad anxiety. I’m always really self-conscious. When I go there, I feel like I can be myself.”*

James, 14

Participants also identified emotional comfort in feeling able to be themselves free from stigma or difficult social situations and furthermore how this facilitated subsequent participation and achievement.

*“It makes me feel comfortable with my own skin. The people are really nice and sweet. It gives me a reason to give all my best.”*

Ashley, 13

Consequent Categories

6. Sense of Mastery and Accomplishment at Learning a New Skill

A sense of mastery, often manifesting in feelings of pride and even amazement at progress made on a surfboard, was frequently reported as integral to participants experiences.

*“I was actually shocked that I did it. I was impressed. I didn’t know I had it in me to be honest.”*

Sarah, 14

This sense of mastery and accomplishment was directly linked with positive outcomes such as confidence primarily through a self-realisation of efficacy or ability based upon their experiences at the interventions and impacting on wider life.

*“Well, I went surfing and I did it. If I can do that then, chances are I can do this thing that’s scary.”*

Jessica 23

*“It’s just something I can look back upon like, “Oh, I did that. I know how to do that and that’s something I’m good at.” It’s a confidence boost.”*

Amanda, 15

7. Enjoying a Sense of Respite/Escape at Beach

Participants often brought up respite and a sense of escapism as another key element to their experience at the Wave Project.

*“I want to do it every week basically because it was just a break from the world for me. I could just chill out and just forget about everything and just have some time to myself and just forget about my worries and that.”*

James, 14

This respite while surfing contrasted with and offered distraction from the difficult situations described in participants wider lives and linked with positive outcomes.

*“It means a lot to me because things that happen at home, different things, it just takes your mind off of it. It’s amazing coming down here. It just makes you forget about everything for a good two hours.”*

Andrew, 12

8. Social Connections with “Surfer” Peer Group

The importance that participants gave to social interactions and development of a new peer groups within the intervention was striking given the prevalence of social anxiety among participants.

*“I love it because I get to meet people who’s going through the same stage as me being all nervous. I can help them go through it.”*

Chris, 14

The intervention setting appeared to increase exposure to, and enjoyment of, the social elements linked to the experience. The intrinsically social nature of the sport seemed to lead to improved self-perceptions of the participant’s ability within a social setting and accordingly led to positive outcomes.

*“I just felt loved again. I felt there was nothing ever going to bring me down anymore because I found loads of new friends. I found people who actually do care and that actually do help me out through my life.”*

Sarah, 14

Additionally, this also led to the development of new peer networks that became a part of participant’s daily support mechanisms.

*“It did translate into my day to day life because I became good friends with people that umm, I started doing more activities with them.”*

Nicole, 18

Example Pathways

While a comprehensive logic model (Figure 3) was developed alongside the identification of core categories, it is important to note that these findings are not prescriptive for individual participant experiences and pathways. In terms of practice, this highlights the importance of making sure all the noted options are available within intervention delivery and not overly focused on individual pathways. While some individuals related to all the categories listed as part of intervention participation, others took more focused pathways which stressed some categories more than others. An example of this could be Jessica whose own experiences were focused the pathways on mastery and respite (top half of Figure 3) with very little discussion of socialisation.

*“It’s like, "Wow I did a thing" and it was alright, and I actually enjoyed it”*

Jessica, 23

*“Actually, it’s a relaxing experience which is nice because I don’t tend to find very many things relaxing.”*

Jessica, 23

This can be contrasted with Michael whose focus was much more to do with respite and social connections with very little discussion of mastery and achievement.

*“It just somehow calms me down so I’m not having to rush too much through my day, so I’m not thinking about other things.”*

Michael, 19

*“It’s much more fun because you’re meeting new people. Because normally I’d be on my own.”*

Michael, 19

This variation of focus is not unforeseen given the wide range of reasons for participant referral and subsequent varied populations within this intervention. What was consistent within the detailed accounts of participant experience was constant relating back to the core categories as part of the intervention process. Furthermore, these core categories converged with the observations of intervention practitioners during the unprompted brainstorming session exploring data saturation. 

## 4. Discussion

Although there is some evidence that surf therapy can be beneficial for mental health, limited research has considered how these outcomes are achieved. This study aimed to explore this knowledge gap by investigating programme theory within the Wave Project surf therapy intervention. Drawing from participants’ experiences, this study developed an in-depth programme theory to help understand the most important elements that enable the intervention to achieve its associated positive outcomes. These findings have the potential to be an important point of reference in the growing field of surf therapy.

Within the programme theory, Antecedent Categories highlight how the intervention operationalises the known inputs to achieve outcomes. In surf therapy, the known inputs include the ocean environment and the trained volunteer instructors. Participants identified that the ocean environment’s intrinsic offering of multiple levels of challenge exists naturally within a surfing sphere, a finding yet to be highlighted in previous literature. The presence of these levels of challenge within programme theory must be highlighted for selection of locations in future surf therapy iterations. While these multiple levels of opportunity occur naturally within the surf setting, they could be replicated within other intervention settings throughout design and implementation. 

The findings furthermore highlight how the Wave Project effectively utilises staff and volunteers in specific ways. There existed within the intervention a clear focus on fostering an atmosphere that rejects concepts of failure and pressure alongside targeted emotional and physical support. These specific actions were fundamental in the creation of a physical and emotional safe space for individuals who may not easily have access to such a space, and in turn was fundamental to the subsequent success of the Wave Project. The general positive impact of peer mentoring within physical activity interventions is well documented, especially in relation to peer support and positive reinforcement [36,37]. 

The core category of Self-Selected Pacing and Progression reflects the importance of participants’ experiences of autonomy in the intervention. This finding reflects current research relating Self Determination Theory (SDT) to physical activity outcomes [38,39,40]. According to SDT, autonomy is a basic psychological need and fulfilment of it enhances psychological well-being whilst also being integral to adherence, enjoyment and continuation of an activity [41]. This description mirrors key experiences identified by participants linking through the entirety of the presented programme theory.

The overarching focus on rejecting failure, removing pressure and providing a degree of autonomy appears to be integral to the success of the programme and a novel combination within a fundamentally skill-based intervention where failure is a naturally inherent element. This approach can be viewed in comparison with examples such as a 2014 sport-for-development football league in Uganda that led to negative mental health outcomes potentially due to the competitive nature of the competition [42].

The findings also suggest that the creation of an emotional and physical safe space was a very important element of participant experience and subsequent mental health improvements. This safe space seems to have been directly facilitated by previously discussed categories related to how volunteers conduct themselves and the removal of concepts of failure while the safe space itself enabled participant experiences of autonomy. Theoretical discussions of safe spaces have occurred across a range of mental health settings [43,44,45]. Safe spaces are often characterised by a lack of judgement with a focus or respect for others and with a psychological sense of community [46]. It must be noted that general participation in surfing outside of intervention settings has not always been identified as a safe space. Several studies highlight how surfing is challenging for beginners, women and people of colour due to a hypermasculine and overtly competitive culture [47,48,49]. This issue was not evident within this study although there could be a range of reasons for this including the age of participants or reliance on the intervention’s continuation programme rather than independent surfing due to own challenges faced. Further focused investigation into transitions from intervention settings to wider surfing culture alongside barriers related to gender and ethnicity could shed more light onto how much of a challenge these present and how to mediate for this. The provision of a safe space in supplement to a skill-based sporting intervention, as opposed to being the focus of an intervention, offers a novel approach to intervention design. This is especially valuable when working alongside at-risk groups or groups that have experienced or are concerned with experiencing stigma. This approach to the provision of safe spaces could be incorporated into existing interventions for their enhancement. 

Three consequent categories provided theoretical pathways from core categories to associated outcomes. Participants consistently reported how perceptions of self-efficacy (the belief that you can successfully accomplish what you set out to do) [50] during surfing directly linked to their own improvements in confidence and self-belief within wider life. This in turn connects this programme theory with the reported positive outcomes associated with the intervention’s success including mental health improvements, confidence and resilience [12]. This pathway is comparable to theoretical discussions of self-efficacy and mastery in other mental health and intervention settings [50,51].

A sense of respite or escape represents a reprieve from negative emotion or symptoms associated with the challenges faced by Wave Project participants and provides a possible pathway to general improvements to mental health associated with the Wave Project [12]. The respite experienced whilst surfing offers hope and belief that positive emotions could exist in other areas of life outside of surfing. This notion of respite is comparable to wider discussions surrounding the ‘distraction’ hypotheses as to why physical activity may enhance mental health [51,52]. In this example, surf therapy goes a step further in not only reducing negative emotions and symptoms, but by also providing a positive alternative. This matches with reported concepts such as fun and happiness being perceived as positive outcomes of participation [12]. Such an experience of respite could be particularly beneficial for those experiencing mental health concerns, as lack of experiencing positive emotions are symptoms of certain conditions, such as major depression or posttraumatic stress disorder. Further exploration of this specific element of the programme theory could be useful within the paradigm of effective positive psychology [53]. 

The findings illustrate the importance of social connections and are consistent with a range of other settings associated with mental health and physical activity [51,54] and are on occasion the core focus of interventions [55]. Once again, this links to the SDT concept of relatedness; the basic human need to have a sense of belonging and connectedness with others [41]. Furthermore, relatedness has been linked to mental health improvements in different settings but with a focus on similar social activities [56] and as an important element of the benefits associated with ‘blue space’ environments [23]. This pathway is somewhat surprising as surfing itself is not inherently social. Other pathways highlighted in this study focus on individual experiences surrounding internal feelings while riding the wave and individual senses of accomplishment. It is important to highlight that the creation of an emotional and physical safe space is fundamental to the successful social element of the Wave Project and represents a novel addition to delivery of an individualistic skill-based intervention. The cited importance of respite and socialisation is an especially interesting finding as both concepts has been previously highlighted as important elements of a separate surf therapy intervention working alongside UK-based military veterans and emergency workers [25,26]. While acknowledging the differing populations targeted, the presence of these previously recognised theories further adds to this programme theory, while also providing initial links from this established body of work to the emergent surf therapy paradigm.

SDT appears to offer a psychological theoretical framework for this programme theory. The findings identify specific categories directly linked with the three basic human needs that SDT ascribes to growth and functioning in competence, relatedness and autonomy [41]. The focus on the promotion of these basic human needs for positive mental health has been used as a framework within a range of different health contexts [57]. The natural presence of SDT’s basic human needs within this intervention signposts a potential framework for further exploration of the surf therapy paradigm while also offering opportunities for future SDT-specific hypotheses testing. 

One notable absence within the identified programme theory was discussion surrounding the impact of the natural ‘blue space’ environment in which surf therapy occurs. Questions exploring the physical environment of the intervention were included in the interview schedule, but the focus remained on the activity and the waves. Furthermore, while some sensory questioning was used in this study, integrating a more embodied approach to specific sensory questioning of participant experience [58] alongside photo-elicitation [59] around “blue space” could lead to better understanding in this area in the future. The lack of discussion may not be surprising given evidence into the relationship between “blue space” and children’s mental health is currently lacking [21] while a stronger link to improved mental health is noted in older adults [23]. Furthermore, it must be noted that the majority of “blue space” discussion is focused on freshwater as opposed to the saltwater environment in which this intervention is based. Despite the lack of discussion surrounding nature and ‘blue space’ within this study, it is possible to hypothesize that as surf therapy participants engage with interventions in the long term, through extended exposure they may come to enjoy the positive benefits associated with ‘blue space’ [16,21,22,23,24]. 

## 5. Implications

The study has provided a grounded programme theory for understanding the theoretical mediators underpinning the Wave Project surf therapy intervention. The mechanistic centred approach of this work led to the recognition of both established and novel theoretical considerations. This work contributes important knowledge and ideas which are likely to have a lasting influence on the practice of surf therapy to tackle mental health burden.

The identification of prominent theories such as mastery, respite and social connectedness is unsurprising given their prevalence in established physical activity and mental health research [51,52,57]. The identification of these theories as plausible and established theoretical mediators lends credence to the Wave Project’s claimed outcomes. 

Some of the theoretical mediators in this study also appear in research surrounding other surf therapy contexts. The presence of similar mediators despite very different participant groups could suggest potential convergence of theoretical underpinnings within the surf therapy paradigm. Surfing is a rapidly emerging sport with a precited global value of $10.3 billion by 2024 [60] with participation across 162 countries worldwide including low- and middle-income settings [61]. The sport, industry and culture are no longer limited to coastal locations due to recent developments in wave pool technology [60]. If potential convergence of theory stands up to further global study, such theoretical understandings could in turn be used to support the operationalisation of surfing within interventions across a wide range of contexts.

The pathways presented in this paper are also of use when it comes to evaluation and service optimisation. While key theoretical mediators have been identified, the antecedent and core categories are of specific use for improving service delivery through training and focus on these categories. For example, the importance of support and removing perceptions of failure/pressure can be highlighted within Wave Project volunteer and staff training to facilitate pathways to improved mental health. Similarly, the importance of multiple challenge levels could be used during site selection for new intervention locations or inform the wave design and operation of emerging artificial wave technologies. This also provides critical process and delivery indicators for evaluation of surf therapy interventions.

While the vehicle of surfing may intrinsically lend itself to enabling these categories, they are certainly not unique to this setting. With this new programme theory, it may be possible to design and optimize programmes within diverse settings, including different physical activity and “blue space” interventions, while still enabling the same pathways to positive mental health outcomes. 

## 6. Limitations

This study applied robust and appropriate research design for the research aims, with rigorous data collection and analysis. The heterogeneous sample was a significant strength of the study offering perspectives of individuals from across England and Wales with an age range of 8 to 23 years and an approximately even gender distribution. Homogeneity was only found in the fact that all individuals had been referred to the Wave Project from professionals working in a variety of contexts. This homogeneity did not expand to exact reasons or diagnoses for referrals, but this reflects the Wave Project’s focus on inclusive referral criteria. This offered an encompassing range of participant experiences within the Wave Project while scope for different experiences not represented in this sample must be accepted.

One of the key aims of this study was to try and understand theoretical mediators within the Wave Project surf therapy intervention through exploration of participant experiences. It must be noted that the study includes some very short interviews. This was namely due to the nature of challenges faced by participants including social anxiety and Autism Spectrum Disorder. On some occasions, despite volunteering to take part in the study, these challenges did lead to shorter descriptions. More time spent interacting with participants at location sites in advance may have helped to mitigate this but unfortunately was not practical or feasible. Despite this, all collected data, including shorter interviews, were analysed with comparison and triangulation offered between the shorter and the more comprehensive descriptions of participant experience. These challenges may also account for some of the shorter quotations reported. The apparent presence of theoretical saturation within the data must be viewed with these challenges in mind, however theoretical saturation was further supported by the informal and unprompted brainstorming session alongside Wave Project practitioners. Further triangulation alongside independent practitioners, parents and teachers would further strengthen the claims of the emergent framework. 

Almost one-third of that sample chose the option for peer support during their interviews. While having another person present during the interview could have facilitated disclosure of content, it also could have limited it based on the relationship with the chosen individual. The option for peer support was deemed an important element in facilitating interviews, especially with younger participants, however it is not possible to know the impact this had on disclosure of content.

Grounded theory practice dictates that initial interview questions are developed based on existing research [27]. This was challenging given the limited nature of theoretical exploration that exists in the surf therapy paradigm with initial questions being generated through practitioner experience alongside comparison to similar grounded studies.

Theoretical mediators for possible physical health outcomes were not explored within this piece of research as they are not a focus of, nor are they reported outcomes within the Wave Project intervention. Future investigation into the relationship between surf therapy and other ‘blue space’ therapies and physical health would be valuable.

No deviant cases, or participants who had dropped off the Wave project intervention were interviewed within this study. Adherence on surf therapy courses within the Wave Project intervention for 2017–2018 was at 78% according to attendance data [62]. Insight as to why 22% do drop off would clarify the limitations of surf therapy in terms of participant experience. Such exploration could be a priority for future studies but was not within the feasible scope for this piece of work. 

## 7. Conclusions

This study took a grounded and participant centred approach to exploring the theoretical mediators within the UK based surf therapy programme, the Wave Project. Through this approach, a programme theory was constructed that allows for in-depth exploration of the processes from known inputs to associated outcomes within the intervention. This programme theory contributes to the emergent evidence base of the surf therapy paradigm highlighting priorities/directions for similar study in different global contexts and offering Self Determination Theory as a possible psychological theoretical framework. Furthermore, these theoretical findings provide pragmatic support for understanding the Wave Project’s delivery, how it can be optimised and further proliferated.

## Figures and Tables

**Table 1:** Different kinds of coding and their purpose within analysis.

Coding Type	Coding Description	Purpose
**Initial**		
Process Coding	Use of gerunds (“ing words”) to describe actions in the data	Used to explore processes associated with change
In Vivo Coding	Direct quotation from the data	Used to directly explore participant perspectives
**Intermediate**		
Focused Coding	Organisation of initial codes into focused categories	Used to extrapolate individual processes and changes into wider conceptual framework and explore interactions
**Advanced**		
Theoretical Coding	Accounts for previous coding within logic modelling	Used to identify core, antecedent and consequent categories demonstrating pathways from known inputs to associated outcomes

## Supplementary Materials

The following are available online at https://www.mdpi.com/1660-4601/16/12/2159/s1, Table S1: Interview Schedule and additional questions added during data collection.
