# Supplementary material for: “When I Go There, I Feel Like I Can Be Myself.” Exploring Programme Theory within the Wave Project Surf Therapy Intervention
Source: Int J Environ Res Public Health. 2019 Jun 18;16(12):2159. doi: 10.3390/ijerph16122159 (PMC6617262; doi:10.3390/ijerph16122159)
Supplement: Supplementary file 1 [file ijerph-16-02159-s001.pdf]

Supplementary Materials

## Interview Schedule and additional questions added during data collection

| **Initial interview Schedule** |
|---|
| Can you tell me about your experiences at the Wave Project? |
| Can you describe to me what it was like meeting with new people at the beach? |
| Can you describe to me how you felt when you caught your first wave? |
| Can you tell me how you felt after the sessions were over? |
| How would you describe yourself as a person at the beach? |
| How, if at all, has surfing impacted on your day to day life? |
| Have you noticed any changes in yourself since you started surfing? |
| Based on your experiences how would you describe the Wave Project to someone who did not know anything about it? |
| **Additional questions added during data collection** |
| Can you tell me what surfing now means to you? |
| Can you describe to me your experiences of the atmosphere at the beach? |
| Can you tell me about working alongside the Wave Project volunteers? |
| Can you describe to me your expectations of surfing prior to starting? (Follow up: How, if at all have these changed?) |
| Can you expand on the emotions you feel at the beach and in the water? |
